# Declining water depth delayed the breeding time of *Fulica atra*, not human disturbance

**DOI:** 10.1371/journal.pone.0202684

**Published:** 2018-08-29

**Authors:** Weiwei Zhang, Tao Liu, Kun Cheng, Paul Rummy

**Affiliations:** 1 Center for Wildlife Resources Conservation Research, Jiangxi Agricultural University, Nanchang, China; 2 College of Wildlife Resources, Northeast Forestry University, Harbin, China; 3 Institute of Vertebrate Paleontology and Paleoanthropology, Chinese Academy of Sciences, Beijing, China; Sichuan University, CHINA

## Abstract

Disturbances by tourists have been considered to delay the breeding time of coots. In this study, we investigated the common coot (*Fulica atra*) from April to June in 2008, 2009 and 2012 around the Anbanghe Nature Reserve and Daqing Longfeng wetland of Heilongjiang Province. We evaluated the correlations of four habitat factors (water depth under coots’ nests, distance of nests to banks, distance of nests to human disturbance and nest coverage) to discuss the impacts of those factors on the breeding time of the coots. The water depth under the nest was significantly correlated with the coots’ breeding stages in the Anbanghe wetland. In addition, we investigated the breeding dates of 56 pairs of coots and found the dates were significantly negatively correlated with the water levels under the nest for both of the wetlands. However, the breeding time (breeding stages and dates) of coots was not significantly related to the distance of the nest to disturbance, distance to the bank of the lake or the nest coverage. The LME models and GAMs that related breeding time to water level received the greatest support. For the GAM, in the group with a clear breeding date, water level was the most influential variable; in the group for which only breeding stages could be recognized, nest coverage combined with water level had a lower AICc value than water depth itself. In conclusion, we found no clear evidence to indicate that disturbances from tourism delayed the breeding time of the coots; however, the water level had a clear influence on the breeding time. We inferred that reproduction was delayed in order to wait for the improvement of habitat conditions (such as food resource and concealment). Neither water level nor disturbance impacted the reproductive output of the coots as these variables showed no clear relationships with the clutch size.

## Introduction

Human activities can cause disturbance events, which can have long-term or short-term influences on the behavior, physiology and breeding of wild animals [1–3]. Disturbances by humans are widely expected to reduce the reproductive fitness of nesting birds [4–6], threaten the habitat suitability and further reduce the sustainability of local populations [7,8], restrict the feeding and breeding chances of wild animals, which could possibly affect the population size instead of simply behavior [9–11], and aggravate the regional extinction of wild species [12–15]. Disturbance events can also potentially affect the timing of breeding in some birds [16,17]. Human activities are a complex disturbance that can affect biodiversity and ecological processes and can vary in frequency, intensity and duration [18]. However, the effects of human activities on wetland-obligated birds might produce a contrasting result [19], and the influence of disturbance is typically measured based on the influences on population size instead of simply on behavior [9,10].

In addition to human activities, waterbirds are also sensitive to disturbance in water factors such as depth, water level fluctuations or the size of the available water area. Waterbird responses to water level can cause fluctuations in population size [20]. To date, existing literature on the impacts of water changes on waterbirds has primarily focused on the abundance or diversity of species, as well as habitat use [21–24]. Water depth variations influence the species composition and the abundance of emergent and submersed vegetation in wetlands [25–28]. The variations also influence the amount of available food, nesting and thermal cover for waterfowl [25, 29].

**Fig 1:**
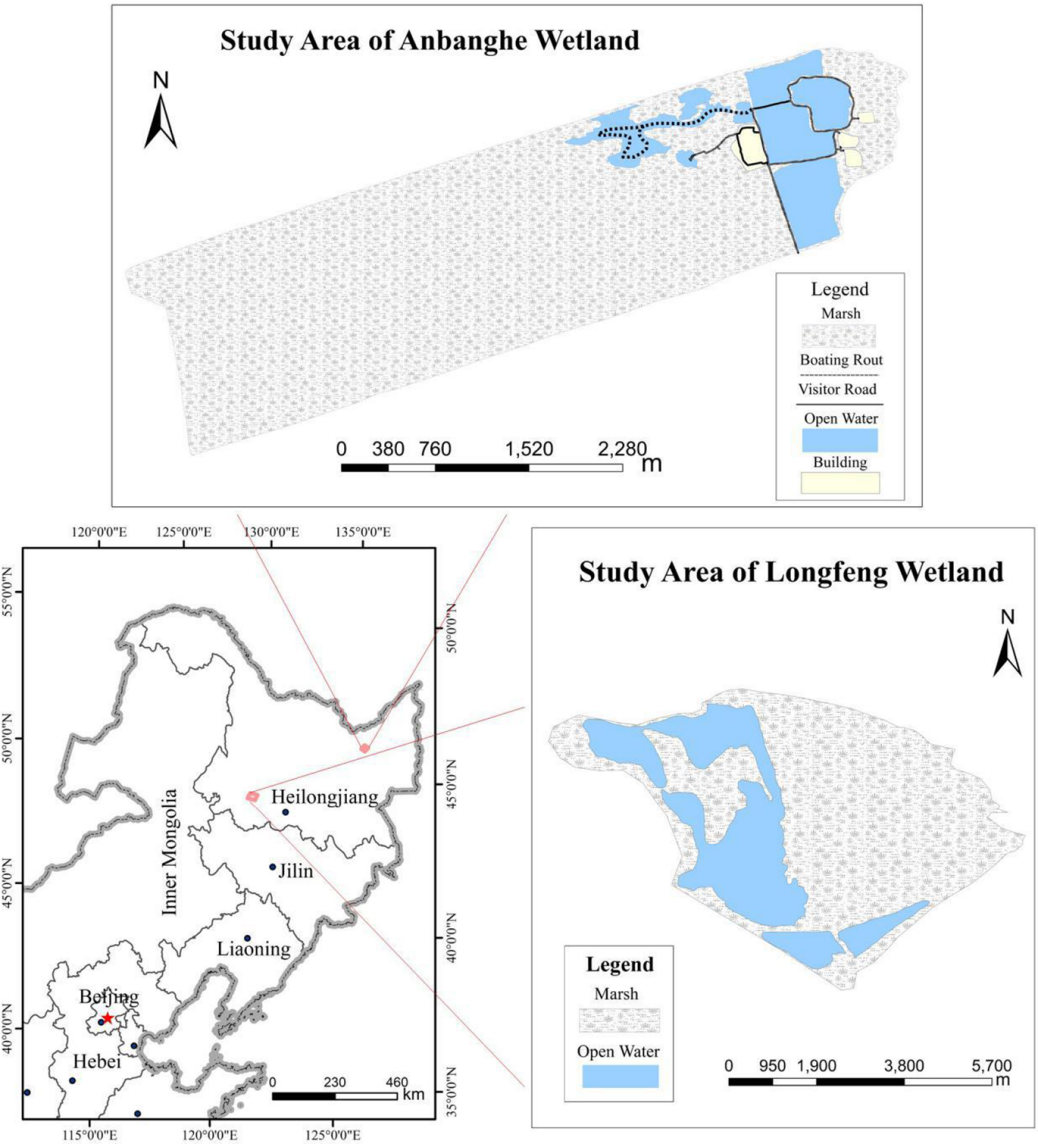
The map of study areas.

The common coot (*Fulica atra*) is a widely distributed waterbird and is found in large numbers in the wetlands of China [30,31]; however, the numbers have recently clearly declined in some areas. For example, in the Zhalong Nature Reserve, which is a national wetland nature reserve in NE China and a major breeding ground for endangered red-crowned cranes (*Grus japonensis*), coots were the dominant species and were found in large numbers [32] in the 1980s. In recent years, however, there has been a sharp decline, and coots are now considered a rare species in this area [32]. The Zhalong Nature Reserve started its ecotourism development activities in the 1990s [17], and a growing number of tourists visit the reserve area. Apart from tourism, the lack of water has also been a key problem in the Zhalong wetland in recent years [33–35]. Tourism exploration and/or water factors may be responsible for the decline in the coot population. The coots in areas open to tourists breed later than those in the tourist-free core areas [17]. This study was carried out in the Anbanghe and Longfeng wetlands in Heilongjiang Province in NE China (see Fig 1). We documented the influence of human disturbance, water level and nest position on the breeding time (including breeding stages and breeding date) of common coots. In addition, we determined the most appropriate management techniques required to minimize these impacts and prevent a population decline of common coots.

## Results

### Factors impacting the breeding time of coots

A total of 218 nests were recorded at the Anbanghe and Longfeng wetlands from 2008 to 2012. The water depth ranged from 16 to 114 cm during the three years (Table 1).

**Table 1:** Nests investigated in two study sites.

		*Anbanghe Wetland*							*LongFeng Wetland*		
Year	No. of nests						Water level (cm) mean±se	Clutch size	No. of nests	Water level (cm) mean±se	Clutch size
2008	Breeding date: 31						65.3 ± 2.065	7.9 ± 0.235 (n = 36)	_	_	_
	Breeding stages: 116	a	b	c	d	e					
		5	12	34	12	53					
2009	18						50.300±4.322	8.40 ± 0.550 (n = 14)	25	56.200 ± 3.540	9.95 ± 0.380 (n = 20)
2012	53						97.968±3.325	8.40 ± 0.259 (n = 57)	_	_	_

**Fig 2:**
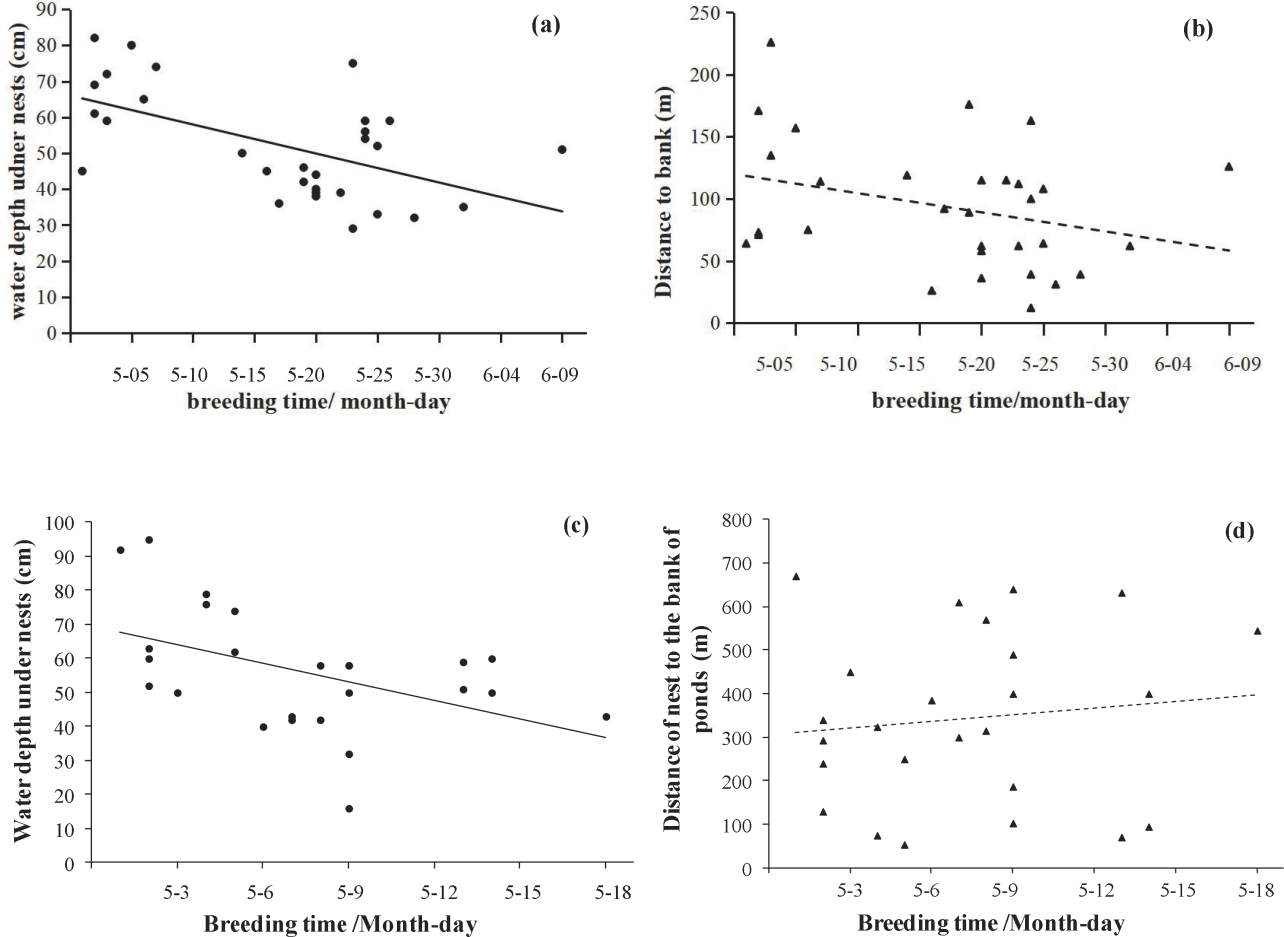
Relationships between breeding date and distance to the banks and water level under nests in Anbanghe wetland (a and b) and Longfeng wetland (c and d).

In the Longfeng wetland, the breeding dates were not correlated with the distance of nests to the bank (*r* = 0.114, *df* = 24, *P* = 0.586) (as shown in Fig 2). However, the breeding dates were significantly correlated with the water depth under the nest (*r* = −0.468, *df* = 24, *P* = 0.018).

Breeding dates and water level were significantly correlated in the two study areas, but there was no significant relationship between breeding time and the distance from the banks (Table 2, Fig 2). An ordinal regression supported a significant correlation of only water level with the breeding stages of coots (*r* = 0.566, *df* = 53, *P* < 0.001).

**Table 2:** Results of LME analyses explaining the probability of coot breeding time and clutch size based on four variables (distance to disturbance, distance of nests to banks, water level under nests, and nest coverage) in the Anbanghe wetland.

Variable	Breeding stage					Breeding date					Clutch size				
	*Adjusted R-squared*	*P*	*AICc*	Δ*AICc*	*wi*	*Adjusted R-squared*	*P*	*AICc*	*ΔAICc*	*wi*	*Adjusted R-squared*	*P*	*AICc*	Δ*AICc*	*wi*
Distance of nests to disturbance (DND)	0.192	0.131	296.75	14.98	0.0006	0.424	0.695	255.72	20.09	0.0000	0.217	0.465	163.91	3.21	0.1044
Distance of nests to the bank of ponds (DNBP)	0.197	0.082	294.32	14.07	0.0009	-0.250	0.617	255.51	19.84	0.0000	0.209	0.966	164.98	4.28	0.0611
Water depth under the nest (WDuN)	0.320	<0.001***	259.49	0	0.9979	0.420	<0.001***	235.63	0	0.9997	0.210	0.811	160.70	0	0.5195
Nest coverage % (NC %)	0.178	0.523	304.86	14.63	0.0007	0.255	0.914	252.54	16.91	0.0002	0.209	0.987	161.69	1.00	0.3151

There was a significant positive impact of water depth under the nest on the coots’ breeding stages, but there were no clear correlations between the breeding stages and the distance of nests to the disturbance or the bank of ponds, as well as nest coverage. Of the three tested factors, only water depth showed a significant correlation with the breeding dates (Table 2). The breeding time seemed earlier as the water level increased.

Models containing disturbance as a variable showed greater AICc values compared with models in which water level was considered separately, and removing the variable of water depth under the nest resulted in a large increase in AICc, suggesting that this was the most influential variable. In the model test results for breeding stages, only nest coverage combined with water level had a smaller AICc than that of the model considering water depth alone; however, in the mixed factor models of the breeding date of coots, the models that contained disturbance based on the distance from nests to the bank of ponds and water level had lower AICc values than the model in which water level was considered alone (Table 2).

### Factors impacting the productivity of the coots

**Fig 3:**
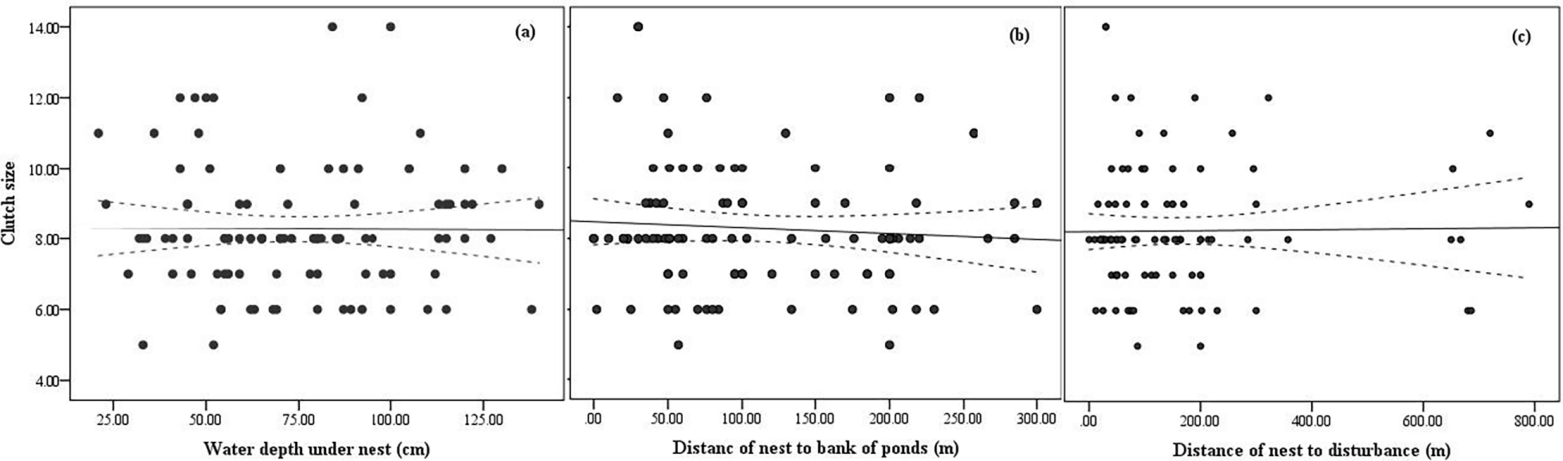
**Correlations showing the relationships between the clutch size with water level, distance of nest to bank of ponds and disturbance of the common coot**s (a: clutch size & WDuN, b: clutch size & DNBP, c: clutch size & DND).

**Fig 4:**
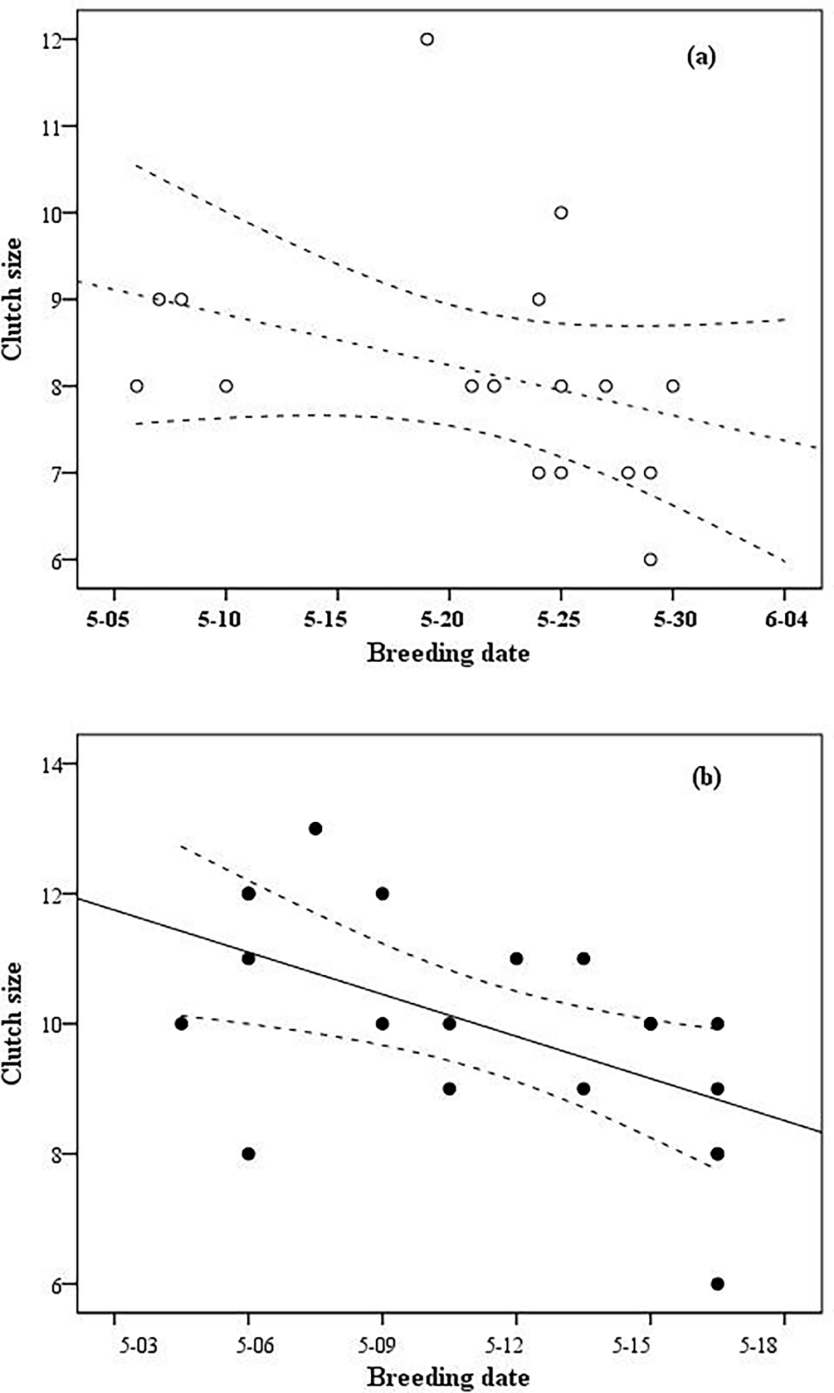
Correlations between the breeding date and clutch size of coots in Anbanghe (a) and Longfeng wetland (b).

We also checked whether the productivity of the coots was influenced by the four studied environmental factors. The results did not support any relation of the studied factors with the clutch size of the coots (see Table 3, Fig 3). The clutch size was only significantly negatively correlated with the breeding date in the Longfeng wetland (see Fig 4, r = -0.420, n = 17, p = 0.012). When we tested whether the clutch size of coots was related to all the variables using generalized additive models, the results still showed no correlations (see Table 3).

**Table 3:** Generalized additive models explaining variation in the factors influencing the breeding time and reproductive output of coots.

Model K	Factors containing				Breeding stage					Breeding date					Clutch size				
	WDuN	DNBP	DND	NC	*Adjusted R-squared*	Deviance explained	*AICc*	Δ*AICc*	*wi*	*Adjusted R-squared*	Deviance explained	*AICc*	Δ*AICc*	*wi*	*Adjusted R-squared*	Deviance explained	*AICc*	*ΔAICc*	*wi*
4	√	√	√	√	0.385	43.0%	259.56	1.88	0.2545	0.451	55.4%	251.14	6.19	0.0235	0.311	41.8%	146.45	2.66	0.0824
2	√	√			0.343	37.0%	259.27	4.55	0.0670	0.417	45.6%	244.95	0	0.5181	0.323	41.1%	144.01	0.22	0.2790
2	√		√		0.334	36.2%	261.63	6.42	0.0263	0.476	54.6%	245.21	0.26	0.4550	0.186	25.4%	147.88	4.10	0.0398
2	√			√	0.379	41.3%	257.15	0	0.6514	0.333	44.4%	256.54	11.59	0.0016	0.279	35.0%	144.28	0.49	0.2437
2			√	√	0.096	12.1%	298.79	39.34	0.0002	0.089	14.0%	260.85	15.91	0.0002	0.190	25.9%	147.78	3.99	0.0424
2		√		√	0.113	13.7%	296.17	37.14	0.0006	0.209	27.0%	256.88	11.93	0.0013	0.340	43.3%	143.79	0	0.3114
3		√	√	√	0.125	15.6%	296.24	36.79	0.0001	0.194	27.9%	259.32	14.37	0.0004	-0.024	3.8%	155.04	11.25	0.0011

DND: Distance of nests to disturbance, DNBP: Distance of nests to the bank of ponds, WDuN: Water depth under the nest (WDuN), NC: Nest coverage

## Discussion

Disturbance delayed the first broods’ breed timing significantly in heather-dominated territories of the Dartford Warbler (*Sylvia undata*) [16]. A similar conclusion was made regarding coots [17], but this hypothesis was not supported by our research. The breeding time was correlated with water depth in both sites whether there was disturbance from visitors or not. Coots laid later when the water depth was shallow.

The coots showed a certain tolerance of direct human disturbance (e.g., visitors and vehicles in the Anbanghe wetland) but were quite sensitive to water level changes [36]. The coots abandoned their nests in the early breeding period when the water level dropped to below 10 cm. We also found that some breeding pairs left their original territory, which might have been caused by the decline in the water level. The tested correlations between the breeding time and the distance from the banks in both sites were nonsignificantly negative. The contrasting results might be influenced by the water level, which was significantly correlated with the distance of the nest to the banks in the Anbanghe wetland but not in the Longfeng wetland (see Table 3). Nevertheless, our results confirmed the influence of water level on the breeding time of coots.

Similar to the American coots [37], the clutch initiation of the common coots delayed as the water depth below the nest decreased. Watermifoil (*Myriophyllum verticillatum*) and hornwort (*Ceratophyllum demersum*) are the main food of coots in the breeding season [38,39]. The growth and biomass of these submerged plants are influenced by water depth [40]. The pecking rates of coots are lower in deeper water than in shallow water and muddy soils [41,42], and the birds choose an appropriate water depth with more food and a lower energy cost for diving. The water level can indicate food abundance, as well as habitat quality to some degree. It appears that a delay in the breeding time of coots can be an adaptive adjustment: the birds wait for the food conditions to improve.

We found insufficient evidence of an impact of the distance to the bank on breeding time and clutch size (see Fig 3). However, in addition to experiencing more disturbance from human activities, breeding pairs inhabiting areas close to the pond margins are always in poorer-quality habitats. Delaying breeding time and waiting for the habitat to improve are positive strategies to improve breeding success and the survival rate of fledglings. However, the breeding time and reproductive output of coots are controlled by the birds’ own conditions to some degree. Coots are territorial birds [43–45]. The pairs that were breeding for the first time generally produced eggs later than other pairs [46]. Weaker birds, or those breeding for the first time, might be at a disadvantage in the competition for territory and, as a result, select poorer habitats to nest, thus resulting in a later breeding time.

The productivity of coots showed a decreasing trend with later breeding dates; however, this trend was significantly supported in only the Longfeng wetland. Similar results were found for American coots [37]. Factors affecting the clutch size of coots are complex and include age, habitat quality (food limitation), laying date, and parasite load [46,47,48]. The coots’ clutch size increased with the increase in the distance from the nest to the edge of the lake [49]. Fledging success of coots is causally related to timing of breeding [50]. The survival rate of the fledgings was influenced by the clutch size [51]. In the Anbanghe wetland and Longfeng wetland, the intrabrood parasitism of coots was severe [48]. In addition, the investigation period was short and insufficient to reveal the impact of water depth on the reproduction of coots over the whole breeding season. Water depth could potentially influence the territory quality due to variations in plant mass and food abundance. Deeper water could be a better barrier against predation by terrestrial mammals. A delay in the breeding date will reduce the chance of a second brood and lead to a later migration from the breeding ground.

In summary, we found tourism activities had limited impacts on the breeding performance of coots in the Anbanghe wetland. Existing studies have shown that the density of American coots (*F*. *americana*) was positively related to water depth [52], and the coots likely preferred high-water wetlands, as they require deep water for foraging and escaping [53,46]. Therefore, we consider that a decrease in water level as well as water surface reduction could be the reasons behind the decline in the coots population. The annual precipitation in the Zhalong wetland has decreased over recent years [32,36], and the construction of a water control project for agricultural irrigation also limited the water supply in the area. Water shortages might cause a reduction in the number of coots in the Zhalong wetland. The disturbance caused by tourism activities can result in behavioral changes in birds. In addition, tourism activities also have an adverse impact on bird populations in the long term, particularly in areas where breeding is concentrated. However, the most important consideration here is the management of water control measures to protect the waterbirds.

## Materials and methods

### Study areas

We investigated the breeding populations of common coots at two sites: Anbanghe Nature Reserve (131°06′12–131°32′24″E, 46°53′07″–47°03′54″N) and Longfeng (125°07′–125°15′E, 46°28′–46°32′N) in Heilongjiang Province. The Anbanghe Nature Reserve is in the north-east part of Heilongjiang Province at the lower reach of the Anbanghe River with a total area of 10,295 ha; the reserve is part of the Sanjiang Plain wetland and has a continental monsoon climate in the temperate zone. The reserve is located in the lower part of a river floodplain wetland and mainly comprises reed swamp habitat. Since 2004, the outer area of the reserve has been developed for ecotourism and provided with recreational water activities, such as boating. The reserve was declared a national wetland park by the State Forestry Administration in 2009 and was categorized as a national scenic area in 2011. The tourist area receives approximately 100,000 individual visits per year and up to 1000 individual visits per day during holidays. The Longfeng wetland, with an area of 5050.39 ha, is also an important wetland for breeding coots that is located 8 km from Daqing city, with relatively few tourism activities there before 2012. We surveyed both sites from late April to late June 2008, April–July 2009 and May–June 2012.

This study was permited by the "Anbanghe Nature Reserve administration”. Longfeng Wetland was open to researchers and visitors, no entry restrictions, as it was not a nature reserve. Even though, we still got the permission from Wildlife Conservation and Nature Reserve Managment Department of Heilongjiang Forstry Department to do field work in these two wetlands.

### Study methods

All the work was done in the field, without direct contact with the study objects, we only observed them, coundted the eggs, and measured habitat factors of their nest place.

As pond banks are often used by walking tourists as visitor routes, and boating has been developed on the extensive open waters in the Anbanghe wetland, we chose the distance from the nest to the pond bank or to the open water surface that was used for boating as the indicator of the degree of disturbance. The water level was human-controlled for boating but still fluctuated according to rainfall. To test the influence of water level on the breeding performance of coots, we checked their breeding status and recorded the date when the nests were checked in three short time segments (within approximately one week; the differences in nest checking date would not change the breeding stage of the breeding coots) from May to June in the Anbanghe wetland in 2008. As the new nests were difficult to find during the short investigation period, resulting in too few new nests to be analyzed, we checked and measured all nests for which the breeding stages could be confirmed to increase the sample size. We classified the breeding stages into five types: (a) nest-building period, indicated by a new nest without eggs, (b) egg-laying period, when the birds had started to lay but not finished (less than 6 eggs), (c) incubation period, with all eggs intact, (d) hatching period, with some eggs hatched but others not hatched, and (e) hatch completion period, indicated by an empty and deep nest without eggs. We used a GPS to plot the positions of the nests, the bank and the open water surface using for boating was drawn by GPS, so we can get the distance of coots’ nest to pen water surface and the pond bank by mesureding them in MapSource software version 6.5. We recorded the breeding date of 31 pairs of coots in Anbanghe wetland in 2008 and of 25 nests in Longfeng wetland in 2009. We estimated the percent coverage of grass (both live and dead) within a 1 × 1 m quadrat centered on the nest as the nest coverage. The distance of the nest to disturbance (road, boating route, and other human activity facilities) and to the bank as well as the water depth under the nest were measured when the nest was found [54], and the clutch sizes of 136 nests in both sites were recorded in 2008, 2009 and 2012. To evaluate the influence of disturbance on the breeding time of the common coots, the Longfeng wetland was investigated as a control region where tourism was absent in 2009.

### Statistical analysis

Following a normality test on the data, the data in line with the normal distribution were subjected to a t-test. Correlation analysis was used to evaluate the relationship between breeding time and the water depth and distance to the bank; a correlation was considered to be significant if the *P* value was less than 0.05. However, tourists always walked along the banks in the Anbanghe wetland, and the water depth becomes shallow close to the bank of pond, so when checking their impacts on breeding performance, we examined interactions between the water depth, distance to the bank, and distance to disturbance using correlation analysis. The statistical significance level was set at *P* < 0.05, and degrees of freedom (df) and *P* values from these models are presented. The means are presented as back-transformed parameter estimates, with the upper and lower 95% confidence limits.

To analyze the impact of environmental factors (water depth under the nest, distance of nest to the bank of ponds, distance of nest to disturbance sources, and the nest coverage) on the breeding time (breeding date: data collected from the nests we can confirm the initiation day of egg laying, and breeding stages: data collected from the nests without clear laying date but the breeding stages could be recognized) and clutch size of coots, we used linear mixed effect (LME) models with a Poisson error structure and a log link function using the lme package and built GAMs in the mgcv 4 package in R (version 3.0.2). We set year as a fixed factor, site as a random factor, and the variables we wanted to test as additional predictors. Ordinal regression was used to test the variable influences on the breeding stage of coots, which is a qualitative variable, and a generalized additive model (GAM) (in R package mgcv) [55] and a POLR model were used to relate the breeding time (breeding date and stages) and reproductive output (clutch size) of coots to the four habitat factors above. AIC was used to test the impact of the parameters on the breeding time and clutch size of the coots, using AICc values [56–58].

## Supporting information

S1 File (ZIP)
